# ZLN005, a PGC-1α Activator, Protects the Liver against Ischemia–Reperfusion Injury and the Progression of Hepatic Metastases

**DOI:** 10.3390/cells13171448

**Published:** 2024-08-29

**Authors:** Celine Tohme, Tony Haykal, Ruiqi Yang, Taylor J. Austin, Patricia Loughran, David A. Geller, Richard L. Simmons, Samer Tohme, Hamza O. Yazdani

**Affiliations:** 1 Department of Surgery, University of Pittsburgh School of Medicine, Pittsburgh, PA 15213, USA; tohmec@upmc.edu (C.T.); haykalt@upmc.edu (T.H.); ruiqiy@upmc.edu (R.Y.); austint4@upmc.edu (T.J.A.); loughranp@upmc.edu (P.L.); gellerda@upmc.edu (D.A.G.); simmonsrl@upmc.edu (R.L.S.); 2 School of Medicine, Tsinghua University, Beijing 100084, China; 3 Center for Biologic Imaging, Department of Cell Biology, University of Pittsburgh, Pittsburgh, PA 15213, USA

**Keywords:** ZLN005, mitochondria, ischemia, reperfusion injury, neoplasm metastasis, hepatocytes, liver, oxidative stress

## Abstract

Background: Exercise can promote sustainable protection against cold and warm liver ischemia–reperfusion injury (IRI) and tumor metastases. We have shown that this protection is by the induction of hepatic mitochondrial biogenesis pathway. In this study, we hypothesize that ZLN005, a PGC-1α activator, can be utilized as an alternative therapeutic strategy. Methods: Eight-week-old mice were pretreated with ZLN005 and subjected to liver warm IRI. To establish a liver metastatic model, MC38 cancer cells (1 × 10^6^) were injected into the spleen, followed by splenectomy and liver IRI. Results: ZLN005-pretreated mice showed a significant decrease in IRI-induced tissue injury as measured by serum ALT/AST/LDH levels and tissue necrosis. ZLN005 pretreatment decreased ROS generation and cell apoptosis at the site of injury, with a significant decrease in serum pro-inflammatory cytokines, innate immune cells infiltration, and intrahepatic neutrophil extracellular trap (NET) formation. Moreover, mitochondrial mass was significantly upregulated in hepatocytes and maintained after IRI. This was confirmed in murine and human hepatocytes treated with ZLN005 in vitro under normoxic and hypoxic conditions. Additionally, ZLN005 preconditioning significantly attenuated tumor burden and increased the percentage of intratumoral cytotoxic T cells. Conclusions: Our study highlights the effective protection of ZLN005 pretreatment as a therapeutic alternative in terms of acute liver injury and tumor metastases.

## 1. Introduction

Liver surgery remains a critical intervention for patients with liver failure or the resection of hepatic metastatic lesions, aiming for therapeutic resolution and improved patient prognosis [1]. However, surgical techniques, including hepatic artery clamping and portal vein occlusion commonly used to minimize blood loss and transfusion, often induce temporary ischemia by disrupting hepatic blood flow [2]. The subsequent reperfusion triggers ischemia–reperfusion injury (IRI), characterized by oxidative stress and activation of inflammatory signaling pathways, exacerbating hepatic tissue damage and facilitating additional tumor metastases [3]. 

Liver IRI is a complex pathophysiological process characterized by distinct ischemic and reperfusion phases. During ischemia, hepatocytes suffer from oxygen deprivation, leading to mitochondrial dysfunction, adenosine triphosphate (ATP) depletion, and subsequent cell death [4,5]. Reperfusion exacerbates injury through ROS generation, triggering a cytokine storm and recruitment of inflammatory cells [4,5]. These events culminate in hepatocellular damage, mediated by the interplay between hepatocytes, resident non-parenchymal cells (NPCs), and infiltrating immune cells [6]. Notably, liver IRI not only induces inflammation and mitochondrial dysfunction but also promotes a pro-tumorigenic environment, characterized by increased cancer cell adhesion, accelerated micrometastasis growth, and heightened recurrence rates following tumor resection [7,8,9].

Devising strategies to curb the pro-inflammatory and pro-tumorigenic effects of liver I/R have the potential to provide significant clinical benefit to cancer patients. We and others have shown that perioperative exercise training has a protective effect against liver IRI [1,10]. Exercise training has been shown to modulate immune system responses, which prevents the formation of a pro-metastatic tumor microenvironment and could potentially enhance immediate postoperative and long-term oncologic outcomes [11,12]. Additionally, exercise has been shown to enhance mitochondrial production in various tissues, as both endurance training and voluntary physical activity can increase the expression of proteins associated with mitochondrial biogenesis and autophagy signaling [13]. Scientific evidence supports the idea that increasing the number of mitochondria in the liver can have a protective effect [14], for the mitochondria play a key role in liver physiology, influencing metabolism, maintaining ROS homeostasis, and ensuring cell survival [15]. Despite the benefits of exercise, it is sometimes unfeasible clinically for patients to undergo a 4–8-week exercise training regimen prior to surgery. Thus, the goal of this study is to use the mechanisms we uncovered through exercise training to create a feasible and alternate pharmaceutical intervention.

ZLN005 (C₁₇H₁₈N₂) is a small benzodiazepine molecule with a molecular weight of 250.34 that can upregulate mitochondrial mass through the PGC-1α biogenesis pathway [16]. Studies have shown that ZLN005 has protective effects in IRI of multiple organs, including the kidney, heart, and brain, as evidenced by reduced endoplasmic reticulum stress and injury in renal ischemia, neuroprotection in cerebral ischemia, and upregulation of antioxidant enzymes with associated decrease in oxidative stress markers in these organs [16,17]. This study aims to investigate the protective effects of ZLN005 against IRI. We hypothesize that ZLN005 will upregulate mitochondrial biogenesis in hepatocytes, concurrently reducing oxidative stress, apoptosis, and acute systemic inflammation, ultimately protecting the liver from IRI and the subsequent I/R-induced metastatic tumor growth in the liver.

## 2. Materials and Methods

### 2.1. Animals 

Male wild-type (C57BL/6) mice (8–10 weeks-old) were purchased from Jackson Laboratories (Bar Harbor, ME, USA). Mice housing was pathogen-free, in a temperature-controlled (21 °C) environment under a 12 h light/12 h dark cycle with free access to food and water. Animal protocols were approved by the Animal Care and Use Committee of the University of Pittsburgh. Moreover, animal research was conducted with the 3R principle formulated by William Russell and Rex Burc.

### 2.2. ZLN005 Treatment 

ZLN005 (MedChemExpress [USA]-HY17538) was first dissolved in dimethylsulfoxide (DMSO, ATCC, Manassas, VA, USA). Our pretreatment regimen consisted of consecutive intraperitoneal (IP) injections of ZLN005 for a short period of time, while previous studies administered ZLN005 orally (12 mg/kg for 4 weeks) [16,18] or IP prior to ischemic stress [17]. To optimize pretreatment duration, we performed a pilot study to compare IP administration for 1, 3, and 6 days. Our data showed significantly reduced tissue injury against hepatic IRI at the 3- and 6-day time points compared to 1 day (Supplementary Figure S1). As no substantial differences were evident between the 3- and 6-day groups, a 3-day IP ZLN005 pretreatment was adopted. Control animals received an equivalent volume of the vehicle (DMSO).

### 2.3. Liver I/R Model 

Following 3 consecutive days of ZLN005 or vehicle pretreatment in mice (n = 10 per group), a standardized nonlethal model of 70% hepatic warm ischemia and reperfusion was induced as previously established by our lab [19,20]. In our model, a 60 min ischemic period along with a 6 h reperfusion was selected based on previous findings demonstrating liver damage at shorter times, poor reperfusion tolerance at longer durations, and peak of inflammatory response [21], providing a reproducible level of hepatic injury for this study. In brief, after a 60 min ischemic period, reperfusion was initiated by unclamping the portal triad artery. Animals were euthanized 6 h post-reperfusion for serum and liver tissue collection. All animals were constantly monitored and were given buprenorphine directly after surgical intervention.

### 2.4. Metastasis Models 

Colorectal liver metastases were induced in ZLN005-pretreated and vehicle mice, as previously described [19]. Briefly, 1 × 10^6^ MC38 cells (murine colorectal cancer cell lines) were injected into the spleen using a 27-gauge needle. Tumor cells were allowed to circulate for 15 min before splenectomy. The mice were then randomly subjected to I/R as described above or control. Mice (n = 5 per group) were constantly monitored and were given buprenorphine directly after surgical intervention and every 6–12 h for the next 72 h. Tumors were allowed to grow for 3 weeks before sacrificing the mice and collecting liver tissue for analysis. 

### 2.5. Liver Damage Assessment in Mouse Liver Samples

Liver damage was evaluated 6 h after the start of reperfusion in all the experiments. Serum levels of alanine aminotransferase (ALT), aspartate aminotransferase (AST), and lactate dehydrogenase (LDH) were measured using the DRI-CHEM 4000 Chemistry Analyzer System (HESKA) [10]. 

For tissue necrosis evaluation, histological liver tissue sections stained with H&E were examined at 40× magnification. The necrotic area was quantified using Image J (Version 1.54j, NIH). The results were presented as the mean of the percentage of necrotic area (mm^2^) relative to the total examined area (mm^2^) [10]. This evaluation was carried out by three independent researchers at different times.

### 2.6. Tumor Burden Analysis 

Three weeks after MC38 injection with or without I/R, the mice were sacrificed, and their livers were harvested. The weights of the liver and the mice were recorded. The tumor area in H&E-stained histological liver sections, viewed at 40× magnification, were quantitatively assessed using Image J (Version 1.54j, NIH). The results were presented as the mean of the percentage of the tumor-occupying area (mm^2^) relative to the total area examined (mm^2^) [10].

### 2.7. Immunofluorescence 

The harvested liver tissue was stored in formaldehyde. The sample sections were incubated with the following primary antibodies: 4-hydroxy-2-nonenal (4HNE) antibody (1:200 Calbiochem, MilliporeSigma, Darmstadt, Germany); COX IV (1:1000 Abcam-ab16056, Cambridge, UK); and CD11b (1:150 Invitrogen, Carlsbad, CA, USA). Moreover, the In Situ Cell Death Detection Kit, TMR red (Millipore Sigma, Mannheim, Germany), was used according to manufacturer’s protocol to determine apoptotic cells.

Hepatocytes plated on coverslips were analyzed in a manner similar to in vivo samples. Following cell fixation and blocking, the cells were incubated overnight with COX IV primary antibody (1:1000 Abcam-ab16056, Cambridge, UK). This was followed by 1.5 h incubation at room temperature with the following secondary antibodies: Alexa Fluor-conjugated Cy3 goat anti-rabbit antibody (1:1000 Invitrogen, Carlsbad, CA, USA) and Alexa Fluor-conjugated 488 phallodin (1:1000 Invitrogen, Carlsbad, CA, USA). Cell nuclei were stained with DAPI. Imaging was conducted using an Olympus Fluoview 1000 microscope, and quantification was performed using NIS Elements software- Version 5.42.01 (Nikon, Tokyo, Japan). All slides were scanned under consistent conditions for magnification, exposure time, lamp intensity, and camera gain. 

### 2.8. Mouse Cytokine/Chemokine 44-Plex Discovery Assay^®^ Array (MD44)

Serum samples (n = 3 per group) were obtained from healthy control, ZLN005, and vehicle-pretreated mice after IRI. The serum samples were sent to Eve Technologies (Calgary, AB, Canada) for chemokine and cytokine analysis, according to manufacturer’s protocol. 

### 2.9. NET Quantification Using MPO-DNA ELISA

A capture ELISA myeloperoxidase (MPO) linked to DNA was performed to quantify neutrophil extracellular traps (NETs) in sera from healthy control mice and in sera from pretreated mice after IRI, as described previously [21]. Briefly, 100 μL of the sample was added to the wells and incubated for 1 h. After three washes, 100 μL of incubation buffer containing a peroxidase-labeled anti-DNA monoclonal antibody (component No. 2, Cell Death ELISAPLUS, Roche; Cat. No. 11774424001) was used. Soluble NET formation was quantified as the percentage increase in absorbance compared to the control. Serum nucleosome quantification was measured using the Cell Death Kit (Roche, Mannheim, Germany).

### 2.10. Primary Hepatocyte Isolation and Treatment 

Mouse hepatocytes were collected from wild-type (C57BL/6) mice. Livers were perfused with perfusion buffer and then digestion buffer according to a published protocol with modification [22]. Briefly, a perfusion buffer made from HBSS without Calcium or Magnesium (Fisher Scientific-14-175-145, Grand Island, NE, USA), EDTA (0.5M-Thermofisher, Grand Island, NE, USA), and HEPES (1M-Thermofisher, Grand Island, NE, USA) was first used, followed by a digestion buffer made of HBSS with Calcuim and Magnesium (Fisher Scientific-14-025-126, Grand Island, NE, USA), HEPES (1M-Thermofisher, Grand Island, NE, USA) and collagenase type 1 (50 mg/mL-Worthington Biochemical, Lakewood, CA, USA), and finally another digestion buffer with HBSS with Calcuim and Magnesium (Fisher Scientific-14-025-126, Grand Island, NE, USA) and HEPES (1M-Thermofisher, Grand Island, NE, USA). The liver was then harvested and scraped into a single-cell suspension. After two low-speed centrifugations (50 × G for 2 min at 6 °C) and wash, mouse hepatocytes were obtained. Percoll (Fisher Scientific-45-001-747, Uppsala, Sweden) was used to eliminate dead cells. Hepatocytes were then plated at 1 × 10^6^ density per well and cultured in Dulbecco’s Modified Eagle Medium (DMEM) (Thermofisher Scientific, Grand Island, NE, USA) with high glucose, L-glutamine, phenol red, 10% fetal bovine serum, and 1% pen strep. 

Primary human hepatocytes were obtained through the Human Hepatocyte Isolation Distribution (University of Pittsburgh), part of the CBRPC, Pittsburgh, Pennsylvania. Human hepatocytes were cultured in DMEM (Thermofisher Scientific, Grand Island, NE, USA) with high glucose, L-glutamine, phenol red, 10% fetal bovine serum, and 1% pen strep. 

For ZLN005 treatment, the cells were pretreated with a 10 µM concentration for 24 h. The cells were then placed in either normoxia (20% O_2_ and 5% CO_2_) or hypoxia (1% O_2_ and 5% CO_2_) for another 24 h, as previously described by Wang et al. [18]. 

### 2.11. Flow Cytometry

Ischemic liver lobes were collected from ZLN005 and vehicle-pretreated mice at 6 h after reperfusion. NPCs were separated from the hepatocytes by two rounds of differential centrifugation (50 rpm for 2 min). The supernatant was further centrifuged (400 rpm for 5 min and two cycles of 1500 rpm for 5 min) to obtain NPCs. Light microscopy confirmed that the NPCs did not contain hepatocytes.

Metastatic livers were harvested 3 weeks after cancer cell injection. The livers were digested with collagenase type IV (Fisher scientific-17-104-019, Grand Island, NE, USA) for 30 min, and NPCs were isolated as mentioned above.

Cell viability was assessed using Zombie violet fixable viability (Biolegend, San Diego, CA, USA). To identify the different immune cells, NPCs were stained with antibodies (BD Biosciences, Franklin Lanes, NJ, USA) against CD45, CD11b, F4/80, Ly6G, CD3, NK1.1, CD4, and CD8 and were ran on the BD LSRII flow cytometer. 

Cultured mouse and human hepatocytes were collected after 24h of hypoxia. Cell apoptosis was assessed using the Propidium Iodide staining solution (BD Pharmigen-Fisher scientific-BD 556547, Grand Island, NE, USA). Mitochondrial mass was quantified using the MitoTracker™ Green FM (Thermofisher Scientific-M7514, Grand Island, NE, USA), according to manufacturer’s protocol. 

All the flow cytometry data were analyzed using FlowJo software (Version 10.10.0).

### 2.12. Quantitative Real-Time PCR

Total RNA was extracted from the liver and cells using the RNeasy Mini Kit (Qiagen, Hilden, Germany) following the manufacturer’s guidelines. mRNA expression levels of genes were measured using SYBR Green two-step, real-time reverse transcription polymerase chain reaction (RT-PCR) [10]. Briefly, 1 μg of total RNA from each sample was used to synthesize cDNA with the cDNA synthesis kit (Takara, San Jose, CA, USA). The PCR reaction mixture was prepared with SYBR Green PCR Master Mix (PE Applied Biosystems, Vilnius, Lithuania). The thermal cycling conditions included an initial 10 min at 95 °C, followed by 40 cycles of 95 °C for 15 s and 60 °C for 1 min, performed on an ABI PRISM 7000 Sequence Detection System (PE Applied Biosystems, Waltham, MA, USA). 

The following mouse primers were used: TOMM20, 5′-GCTAAGGAGAGAGCTGGGCTTT-3′ 5′-TGGTCCACACCCTTCTCGTAGT-3′. TFAM, 5′-GAGGCAAAGGATGATTCGGCTC-3′ 5′-CGAATCCTATCATCTTTAGCAAGC-3′. PGC-1α, 5′-AGCCGTGACCACTGACAACGAG-3′ 5′-GCTCATGGTTCTGAGTGCTAAG-3′. NRF1, 5′-CTGCTGTCTCTTTCGGATAGATC-3′ 5′-CGGAAACGGCCTCATCTCT-3′. COXI, 5′-GCCCCAGATATAGCATTCCC-3′ 5′GTTCATCCT GTTCCTGCTCC-3′. β-actin, 5′-GCTCTTTTCCAGCCTTCCTT-3′ 5′-TGATCCACATCTGCTGGAAG-3′.

### 2.13. mtDNA Count Quantification

Total DNA was extracted from the liver using the DNeasy Blood and Tissue Kit (Qiagen, Hilden, Germany) following the manufacturer’s guidelines. Mitochondrial DNA (mtDNA) levels were quantified using SYBR Green two-step RT-PCR. The PCR reaction mixture was prepared with SYBR Green PCR Master Mix (PE Applied Biosystems, Vilnius, Lithuania). The thermal cycling conditions included an initial 2 min at 50 °C followed by 10 min at 95 °C followed by 40 cycles of 95 °C for 15 s and 60 °C for 1 min on an ABI PRISM 7000 Sequence Detection System (PE Applied Biosystems, Waltham, MA, USA) [23]. 

The following mouse primers were used for mMITO, 5′-CTAGAAACCCCGAAACCAAA-3′ 5′-CCAGCTATCACCAAGCTCGT -3′. HK2, 5′-GCCAGCCTCTCCTGATTTTAGTGT-3′ 5′-GGGAACACAAAAGACCTCTTCTGG-3′. COXI, 5′-GCCCCAGATATAGCATTCCC-3′ 5′GTTCATCCT GTTCCTGCTCC-3′. β-actin, 5′-GCTCTTTTCCAGCCTTCCTT-3′ 5′-TGATCCACATCTGCTGGAAG-3′.

### 2.14. Cytochrome c ELISA

To measure cytochrome c levels, the supernatant was collected from cultured mouse and human hepatocytes in normoxia and in hypoxia. A Cytochrome c ELISA Kit (Abcam, Waltham, MA, USA) was used according to the manufacturer’s protocol.

### 2.15. Statistical Analyses 

All statistical analyses were performed on GraphPad Prism 10.1.0. The initial normality of the distribution was assessed using the D’Agostino–Pearson test (alpha = 0.005) and the Shapiro–Wilk test (alpha = 0.005). After passing the normality, group comparisons were performed using ANOVA and pairwise comparisons using Student’s *t*-test. A * *p* < 0.05, ** *p* < 0.01, *** *p* < 0.001, and **** *p* < 0.0001 were considered statistically significant. The results are expressed as the mean ± standard error of mean (SEM). 

## 3. Results

### 3.1. ZLN005 Pretreatment Decreases Liver Ischemia–Reperfusion Injury

**Figure 1:**
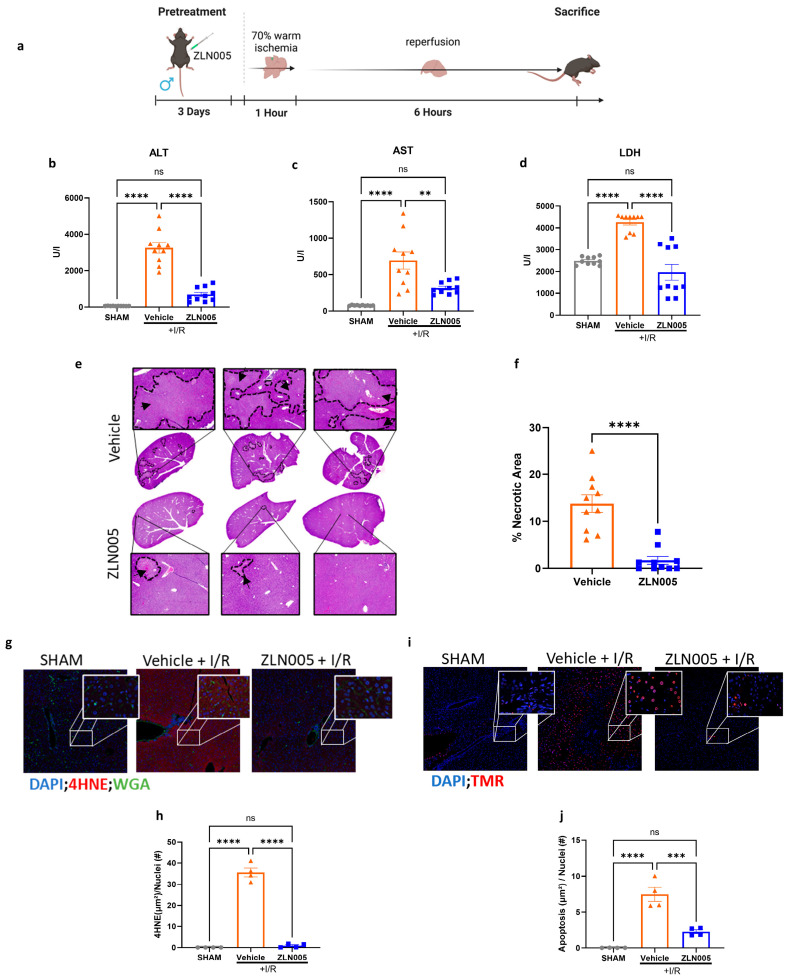
ZLN005 pretreatment decreases liver ischemia–reperfusion injury. (**a**) A schematic representation of the liver I/R mouse model: The mice were pretreated for 3 consecutive days with ZLN005 (12 mg/kg) or with the vehicle intraperitoneally, followed by liver I/R to prompt surgical stress. (**b**–**d**) Serum ALT, AST, and LDH were measured after 1 h of ischemia and 6 h of reperfusion. (**e**,**f**) Representative H&E-stained liver sections and quantification indicating reduced areas hepatic necrosis in mice pretreated with ZLN005 following IRI (areas pointed to by the black arrows and the dotted lines show necrotic tissue, while the rest are normal tissue.). (**g**,**h**) Confocal microscopy immunofluorescence images and quantification of liver sections of mouse liver sections showing decreased ROS production in livers of mice pretreated with ZLN005 compared to those pretreated with the vehicle following IRI. (4HNE shown in red, wheat germ agglutinin shown in green, and nuclei shown in blue). (**i**,**j**) Confocal microscopy immunofluorescence images and quantification of mouse liver sections showing decreased cell apoptosis in the livers of mice pretreated with ZLN005 compared to those pretreated with the vehicle following IRI (TMR shown in red and nuclei shown in blue). All the data are represented as mean ± SE. Statistical comparisons were performed using one-way ANOVA and Student’s *t*-test (**f**) with (****) indicating *p* < 0.0001, (***) indicating *p* < 0.001, (**) indicating *p* < 0.01, and (ns) indicating not significant.

We have previously shown that exercise training protects the liver against IRI [10], and that this protection is through mitochondrial biogenesis upregulation [24]. Therefore, we sought to test whether ZLN005, a PGC-1α activator, would confer protection against liver IRI (Figure 1a). Mice pretreated with ZLN005 showed significantly decreased liver injury as evident by reduced ALT, AST, and LDH serum levels (Figure 1b–d). Tissue histology were consistent with the serum findings, showing significantly decreased hepatocellular necrosis in ischemic lobe of ZLN005-pretreated mice compared to the vehicle (Figure 1e,f). Concurrently, decreased oxidative stress in the ZLN005-pretreated group were observed, as measured by 4HNE staining indicating cellular ROS accumulation (Figure 1g,h). Additionally, tissue immunofluorescence staining of TMR, a marker for cell apoptosis, was significantly decreased in liver tissues from the ZLN005-pretreated group (Figure 1i,j).

### 3.2. ZLN005 Decreases Acute Systemic and Intrahepatic Inflammatory Response to Liver I/R

**Figure 2:**
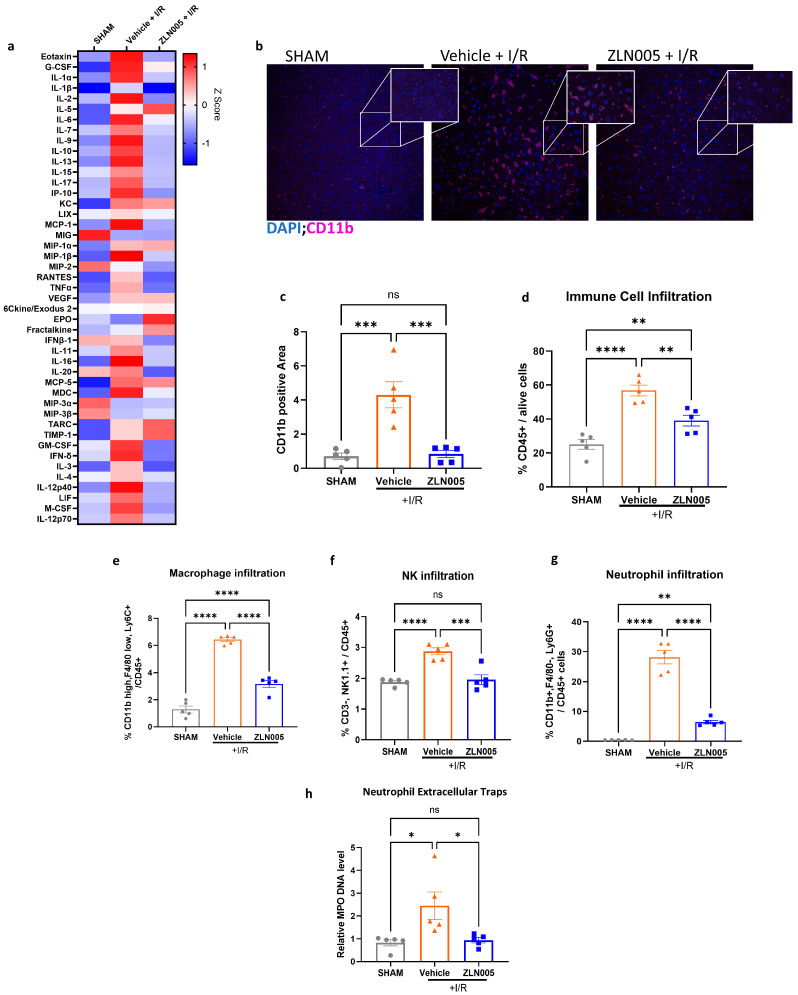
ZLN005 decreases acute systemic and intrahepatic inflammatory response to liver I/R. (**a**) A Luminex assay analysis of serum pro- and anti-inflammatory cytokines. (**b**,**c**) Confocal microscopy immunofluorescence images and quantification of liver sections showing decreased CD11b staining in livers of mice subjected to liver I/R and pretreated with ZLN005 compared to the vehicle. (CD11b shown in purple and nuclei shown in blue). (**d**–**g**) Flow cytometry analysis of hepatic cells after ischemia and 6 h of reperfusion revealing a significant decrease in total CD45^+^ cells, macrophages, natural killer cells (NK), and neutrophil populations in ZLN005-pretreated mice compared to the vehicle. (**h**) MPO-DNA ELISA results showing a decrease in NETs levels in the serum of mice pretreated with ZLN005 and subjected to liver I/R compared to the vehicle. All data are represented as mean ± SE. Statistical comparisons were performed using one-way ANOVA with (****) indicating *p* < 0.0001, (***) indicating *p* < 0.001, (**) indicating *p* < 0.01, (*) indicating *p* < 0.05, and (ns) indicating not significant.

It is well established that liver IRI is mediated by a pro-inflammatory immune response involving immune cell infiltration and cytokine release that culminates in cell injury [24]. Since ZLN005 pretreatment decreased liver injury after I/R, we next sought to investigate the local and systemic immune response in these mice. The Luminex assay revealed a significant increase in pro-inflammatory cytokines in vehicle-pretreated mice subjected to liver I/R but not in the ZLN005 group. We next investigated the immune cell populations in these livers. Tissue immunofluorescence revealed that livers harvested from ZLN005-pretreated mice displayed a significantly decreased proportion of cells positive for CD11b, a marker for innate immune cells such as macrophages and granulocytes (Figure 2b,c) [25]. Consistent with these findings, flow cytometry analysis of ZLN005-pretreated hepatic NPCs showed a significant decrease in proportion of CD45^+^ immune cells (Figure 2d). Examining the various immune cell populations further revealed a decrease in the infiltration of macrophages (CD11b^+^ F4/80^+^), NK cells (CD3^−^ NK1.1^+^), and neutrophils (CD11b^+^ Ly6G^+^) in the ZLN005-pretreated livers 6 h post-reperfusion (Figure 2e–g). Furthermore, NET formation after liver I/R was also significantly decreased in ZLN005-pretreated livers, as evident by the low serum MPO-DNA levels (Figure 2h).

### 3.3. ZLN005 Pretreatment Upregulates the Hepatic Mitochondrial Biogenesis Pathway 

**Figure 3:**
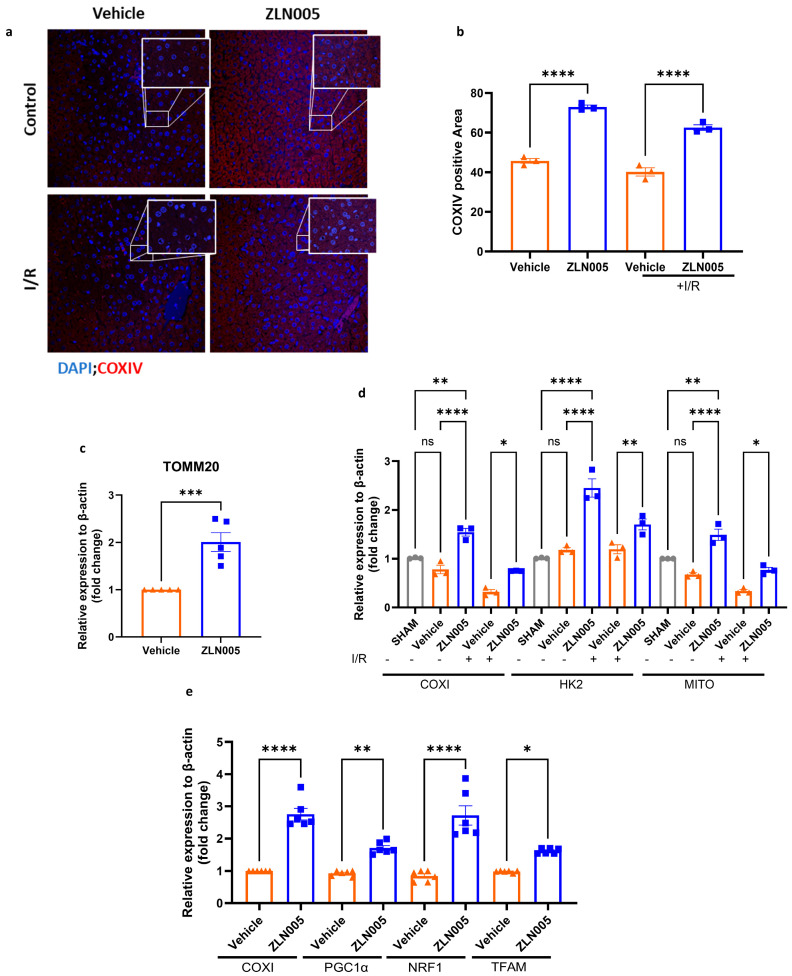
ZLN005 pretreatment upregulates the hepatic mitochondrial biogenesis in vivo. (**a**,**b**) Confocal microscopy immunofluorescence images and quantification of liver sections showing increased COXIV staining intensity in the livers of mice pretreated with ZLN005 compared to the vehicle (COXIV shown in red and nuclei shown in blue). (**c**) qRT-PCR analysis of mRNA showing an upregulation of TOMM20 gene expression in the liver tissue of ZLN005-pretreated mice compared to the vehicle. (**d**) qRT-PCR analysis of total DNA showing amplification of COXI, HK2, and MITO genes belonging to mtDNA in the liver tissue of ZLN005-pretreated mice compared to the vehicle, at baseline and maintained after liver I/R. (**e**) qRT-PCR analysis of mRNA showing upregulation of COXI, PGC-1α, NRF1, and TFAM gene expression in the livers of ZLN005-pretreated mice compared to the vehicle. All data are represented as mean ± SE. Statistical comparisons were performed using one-way ANOVA and Student’s *t*-test (**c**) with (****) indicating *p* < 0.0001, (***) indicating *p* < 0.001, (**) indicating *p* < 0.01, (*) indicating *p* < 0.05, and (ns) indicating not significant.

We have shown that ZLN005 pretreatment can diminish liver IRI and attenuate the pro-inflammatory microenvironment, which is considered a hallmark of IRI. We next investigated whether ZLN005 pretreatment activated the hepatic mitochondrial biogenesis pathway. Indeed, immunofluorescence staining of COXIV in liver tissue showed a significant increase in the mitochondrial mass in mice pretreated with ZLN005 compared to the vehicle. Additionally, this increase in mitochondrial mass was preserved and remained elevated after liver I/R (Figure 3a,b). In addition, tissue mRNA levels of TOMM20 were significantly increased, indicating an abundance of mitochondria in the ZLN005-pretreated group before liver I/R (Figure 3c). To further validate our findings, we measured the mitochondrial DNA content by quantifying the counts of mitochondrial genes such as COXI, MITO, and HK2 [26]. This revealed a significant upregulation of mitochondrial DNA in the ZLN005-pretreated livers, which is also conserved after I/R (Figure 3d). In addition, we investigated the activation of the COXI-PGC-1α biogenesis pathway by qRT-PCR of target genes [27,28] and observed that ZLN005 pretreatment significantly increased the hepatic expression of genes in this pathway, including TFAM and NRF1 (Figure 3e) prior to liver I/R. 

**Figure 4:**
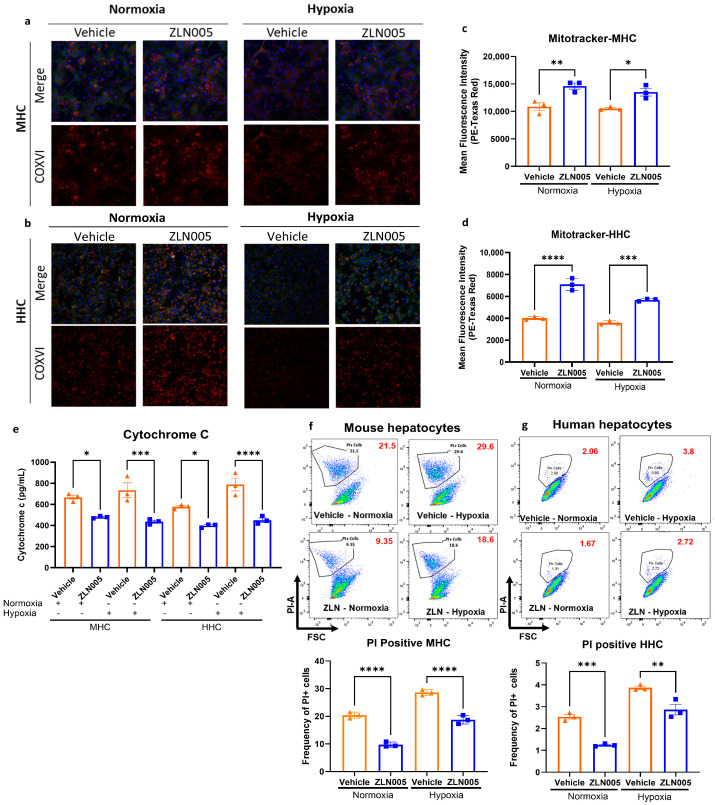
ZLN005 treatment increases mitochondrial mass in hepatocytes and decreases cell apoptosis in vitro. (**a**) Confocal microscopy immunofluorescence images of MHC showing increased COXIV staining intensity after ZLN005 treatment (COXIV shown in red, actin shown in green, and nuclei shown in blue). (**b**) Confocal microscopy immunofluorescence images of HHC showing increased COXIV staining intensity after ZLN005 treatment. (COXIV shown in red, actin shown in green, and nuclei shown in blue). (**c**) Flow cytometry analysis of Mitotracker Green staining in MHC showing increased mitochondrial mass after ZLN005 treatment. (**d**) Flow cytometry analysis of Mitotracker Green staining in HHC showing increased mitochondrial mass after ZLN005 treatment. (**e**) Quantification of cytochrome c release by ELISA from MHC and HHC, showing significantly reduced levels in MHC and HHC treated with ZLN005. (**f**) Flow cytometry analysis of PI^+^ MHC showing fewer apoptotic cells in the ZLN005-treated group. (**g**) Flow cytometry analysis of PI^+^ HHC showing fewer apoptotic cells in the ZLN005-treated group. All data are represented as mean ± SE. Statistical comparisons were performed using one-way ANOVA with (****) indicating *p* < 0.0001, (***) indicating *p* < 0.001, (**) indicating *p* < 0.01, and (*) indicating *p* < 0.05.

Since hepatocyte dysfunction and death is the initial driver of hepatic injury after I/R, we next sought to investigate the effect of ZLN005 on hepatic mitochondrial levels and stress response in isolated hepatocytes from both mouse and human tissue samples. Consistently, the in vitro studies showed a significant increase in mitochondrial mass in both mouse hepatocytes (MHC) and human hepatocytes (HHC) after ZLN005 treatment for 48h, evident from COXIV immunofluorescence staining (Figure 4a,b and Supplementary Figure S2), and Mitotracker flow cytometry analysis (Figure 4c,d). Additionally, cell survival under hypoxia was improved in the ZLN005-treated hepatocytes compared to the vehicle, as evident by the decrease in cytochrome c release (Figure 4e). This was also shown via flow cytometry analysis of PI^+^ hepatocytes, which indicated more prominent cell injury in the vehicle-treated hepatocytes compared to the ZLN005-treated hepatocytes (Figure 4f,g). Interestingly, ZLN005 treatment further enhanced hepatocytes’ survival under normoxic conditions, as hepatocytes tend to undergo apoptosis in culture even under normal conditions [22,29].

### 3.4. ZLN005 Protects the Liver against Liver IRI-Induced Tumor Metastasis 

**Figure 5:**
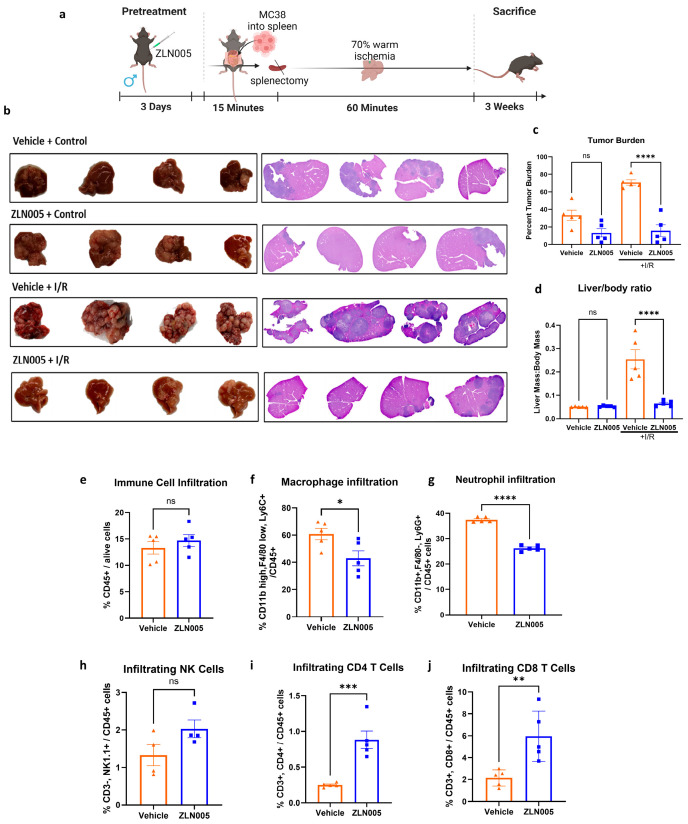
ZLN005 protects the liver against liver IRI-induced tumor metastasis. (**a**) A schematic representation of the liver metastasis mouse model: Mice were pretreated for 3 consecutive days with ZLN005 (12 mg/kg) or with the vehicle intraperitoneally and were then subjected to splenic injection of 1 × 106 MC38 cells, which were allowed to circulate for 15 min, followed by splenectomy with or without 1h of liver ischemia. (**b**) Representative images of gross tumor along with H&E staining 3 weeks after liver collection from control and I/R groups pretreated with ZLN005 or the vehicle. (**c**) Quantification of tumor burden showing a significant decrease in IRI-induced metastatic tumor growth after ZLN005 pretreatment. (**d**) Ratio of liver to body mass showing a significant decrease in IRI-induced metastatic tumor growth after ZLN005 pretreatment. (**e**–**j**) Flow cytometry analysis of hepatic tumor microenvironment after IRI revealing no significant change in total CD45^+^ cells and NKT cells, a significant decrease in macrophage and neutrophil infiltration, and a significant increase in CD4^+^ T cells and CD8^+^ T cells in mice pretreated with ZLN005 compared to the vehicle. All data are represented as mean ± SE. Statistical comparisons were performed using one-way ANOVA (**c**,**d**) and Student’s *t*-test with (****) indicating *p* < 0.0001, (***) indicating *p* < 0.001, (**) indicating *p* < 0.01, and (*) indicating *p* < 0.05.

Given that the inflammatory environment of liver IRI has been shown to promote metastasis formation [9], we next evaluated whether ZLN005 can protect against the development of tumor metastases in the perioperative period. Using our well-established tumor metastasis model (Figure 5a), we observed no difference in tumor growth between the vehicle and ZLN005-pretreated control groups. However, the tumor burden was significantly reduced in the ZLN005-pretreated group after I/R compared to the vehicle, as evident by images of hepatic tumors, tumor burden, and the liver to body weight ratio (Figure 5b–d). Investigation of the immune cell infiltration into these tumors revealed a significant decrease in the macrophages, neutrophils, and NK cells, paralleled with an increase in CD4^+^ and CD8^+^ T cells in ZLN005-pretreated groups (Figure 5e–j).

## 4. Discussion

Liver IRI not only contributes to acute liver injury and delayed graft function but also plays a role in the metastatic process, particularly in cancer patients undergoing major liver resection. The cellular damage, characterized by inflammatory response, oxidative stress, and mitochondrial dysfunction during the reperfusion phase can create a favorable environment for tumor establishment and the growth of metastases. Therefore, developing effective strategies to mitigate IRI is crucial. In this study, using a mouse model of hepatic IRI and metastases, we show that a 3-day preconditioning of the liver with ZLN005, a PGC-1α regulator, prior to I/R was associated with significant decreases in liver injury and I/R-induced metastatic progression. This protective effect was predominantly mediated through an increase in mitochondrial mass via the mitochondrial biogenesis pathway, resulting in decreased oxidative stress, inflammatory cell response, and enhanced hepatocellular protection. In addition, ZLN005 pretreatment markedly decreased I/R-induced hepatic tumor burden, thus enhancing long-term oncological outcomes. 

Given that dysfunctional mitochondrial health is a critical pathological feature of aging and various diseases [26], it is important to recognize the pivotal role of mitochondrial health in maintaining cellular function, particularly in organs with high energy demand such as the liver. Mitochondria are responsible for generating ATP through oxidative phosphorylation, efficiently managing ROS levels to prevent oxidative stress, protecting cellular components, and regulating key metabolic pathways including fatty acid oxidation and glucose metabolism [27]. During warm liver IRI, hypoxia increases hepatic ROS production [28], which is exacerbated upon reoxygenation. These changes significantly disrupt the mitochondrial metabolic flux, further potentiating oxidative stress and contributing to hepatocellular apoptosis and tissue necrosis. Our results show that a short-term ZLN005 pretreatment, administered both in vivo and in vitro, significantly reduced ROS and its downstream release of cytochrome c. This resulted in improved hepatocyte survival, demonstrated by reduced apoptosis in injured liver tissue and cultured human and mouse hepatocytes. Furthermore, ZLN005 treatment increased mitochondrial mass as evident by the upregulation of the mitochondrial biogenesis and the mitochondrial DNA count. These findings correlated with decreased levels of ALT, AST, LDH, and tissue necrosis suggest a potential link between restored mitochondrial function and protection against IRI. This notion is corroborated by Rui et al., who demonstrated a strong correlation between hepatic mitochondrial function and I/R in 28 liver transplant patients [30]. Furthermore, our finding suggests that while IRI typically results in a loss in mitochondrial mass, ZLN005 pretreatment significantly reduces this loss by generating more mitochondria, indicating that ZLN005 may help revert mitochondrial-induced tissue injury. It would be valuable to further investigate the effect of introducing ZLN005 during the reperfusion phase of IRI, as opposed to pretreatment. This is particularly relevant given that a previous study by Xu.et al. showed that a single dose of ZLN005, given four hours after ischemia onset, was effective in reducing brain infarct volume and improving neurological deficits [17]. 

In addition to mitigating mitochondrial injury, the treatment with ZLN005 also appears to have a broader impact on inflammatory response. During liver IRI, mitochondrial injury is known to cause an abundant release of DAMPs and cytokines from hepatocytes and local immune cells into liver tissue systemically [31]. This cytokine storm was significantly attenuated in mice pretreated with ZLN005, as evident by a significant decrease in several pro-inflammatory cytokines. Notable among these are MCP-1, TNFα, IL-12p40, MIP1β, RANTES, IL-6, G-CSF, IL-7, IL-16, 1L-1α, IL-3, IL-2, MDC, MCSF, IL-9, IL-17, EOTAXIN, IP10, LIF, IL-12p70, IL-15, GM-CSF, LIX, IL-11, IFNδ, and IL-20. MCP-1 or monocyte chemoattractant cytokine-1 is responsible for regulating monocyte and macrophage infiltration [32], and TNFα promotes neutrophil chemotaxis into inflamed tissues by upregulating their adhesion proteins and enhancing their rolling, binding, and parenchymal extravasation. TNF-α has also been shown to increase the release of other pro-inflammatory cytokines, including IL-6 and GM-CSF [6]. Conversely, the anti-inflammatory cytokine erythropoietin (EPO) was upregulated in the ZLN005-pretreated group. EPO has previously been shown to limit the pro-inflammatory microenvironment in liver IRI [33]. These observations suggest that ZLN005 pretreatment either mitigates systemic inflammation or exerts peripheral effects on the immune system, leading to an attenuated inflammatory response after liver IRI. Overall, these pro-inflammatory and pro-tumorigenic events occurring in the liver are known to be exacerbated in dysfunctional mitochondria and were significantly attenuated by a pretreatment course of ZLN005, a mitochondrial biogenesis activator.

The concept of enhancing cellular mitochondrial fitness through tissue preconditioning has emerged as a promising yet relatively underexplored strategy for mitigating stress response. Experimental studies have shown that mitochondrial transplantation can protect against various diseases and injuries, including traumatic brain injury, oxidative stress, neuron apoptosis following IRI, cardiac remodeling and apoptosis, and improving cardiac function [34,35]. Additionally, mitochondrial transplantation has proven beneficial in conditions such as inflammatory cytokine modulation in macrophages, sepsis-induced mitochondrial dysfunction, acute kidney injury, liver injury and fibrosis, and reduction in IRI in lung transplantation [34]. In our current study, we demonstrated that an upregulation of mitochondrial biogenesis through ZLN005 administration can reduce ROS production, cellular apoptosis at the injury site, and liver injury and necrosis. Furthermore, ZLN005 was found to have a direct effect on hepatocytes in vitro, suggesting its potential efficacy regardless of the administration method. This finding is particularly relevant for future studies exploring the use of ZLN005 during organ perfusion to enhance hepatocytes’ survival and health prior to liver transplantation. Previous studies have shown that in vitro mitochondrial transplantation can be a viable option for patients with liver failure, liver injury, or liver transplantation [36]. Supporting this approach, Wang et al. have demonstrated that intravenous injection of an adenoviral vector to overexpress PGC-1α, a crucial activator of mitochondrial biogenesis, 3 days prior to liver I/R significantly decreased liver injury, ROS production, and tissue inflammation [37]. Similar protective effects have been observed in models of cerebral and cardiac ischemia [38,39]. Additionally, other compounds, such as honokiol, which act similarly to ZLN005 by targeting PGC-1α, have shown promise in reducing neuronal ischemia–reperfusion injury [40]. 

To further contextualize these findings, our previous work demonstrated that exercise training increases hepatic mitochondrial mass, rendering the liver more resistant to damage during IRI [10]. Specifically, we observed that 4 weeks of exercise training, achieved through daily treadmill running in mice, decreases liver IRI- and I/R-induced hepatic metastatic growth [10], likely through the modulation of hepatic metabolism and upregulating PGC-1α activity. In this manuscript, we focus on studying the in vivo and in vitro effects of ZLN005, a specific PGC-1α transcriptional activator, to circumvent the limitations of exercise interventions and potential off-target effects of pharmacological alternatives. As a master regulator of mitochondrial metabolism, PGC-1α activates several OXPHS genes and enhances mitochondrial fitness and mitochondrial biogenesis through upregulating NRF-1/2 and TFAM, leading to increased replication of mitochondrial DNA [41]. The increase in PGC-1α, NRF1, and TFAM levels, as well as mitochondrial DNA count after ZLN005 treatment, indicate a robust activation of mitochondrial biogenesis induced by ZLN005 pretreatment. By increasing PGC-1α levels, ZLN005 improves the mitochondrial lifecycle and the cellular stress response to IRI. Given the established link between liver IRI and tumor metastasis [42,43], ZLN005-mediated IRI protection is logically a natural explanation for the attenuation of metastatic tumor growth in the liver that we have observed in our current study. However, other factors also need to be considered, as the direct effect of ZLN005 on cancer cell behavior has not been studied in the literature. Studies have shown that activating mitochondrial biogenesis in cancer cells using osteopontin may increase tumor metastasis [44]. In addition, further studies are needed to examine how altering the mitochondrial dynamics in immune cells modulates the delicate balance of anti-tumorigenic and pro-tumorigenic immune responses in the tumor microenvironment [45]. 

Although the in vivo effects of ZLN005 are still incompletely understood, previous studies have showed that systemic administration of ZLN005 through oral gavage or intraperitoneal injections can upregulate mitochondrial biogenesis in other organs, such as the kidney, skeletal muscle, heart, and blood vessels and can protect vital organs, such as the kidney and brain, against IRI in experimental models [16,17,18]. We acknowledge that further studies are needed to examine the dependency of the protective effects of ZLN005 on hepatic PGC-1α and to elucidate the complete molecular effects of ZLN005 on various other cell types within the liver microenvironment or in different tissue compartments. Moreover, ZLN005 has been shown to increase fat oxidation and improve glucose tolerance, pyruvate tolerance, and insulin sensitivity in diabetic mice [16], and suppress hyperglycemia-induced cardiomyocyte injury [46]. Additionally, ZLN005 can ameliorate unilateral ureteral obstruction-induced renal fibrosis [47], improve the immune response in early polymicrobial sepsis [48], and decrease pathogenicity of P. aeruginosa infection [49], among other reported effects. We believe that by increasing mitochondrial biogenesis and reducing the pro-inflammatory environment, ZLN005 improves the downstream stress responses which would then mitigate the progression of various diseases including tumor progression.

We acknowledge that our current study is limited in investigating the effect of ZLN005 on hepatocytes in the context of IRI. While we observed a significant increase in mitochondrial mass within hepatocytes, a more comprehensive analysis of individual NPC populations, including Kupffer cells, endothelial cells, and hepatic stellate cells, is warranted given their critical roles in liver IRI pathogenesis. Additionally, a phenotypic characterization of infiltrating immune cells was not performed. Our previous work demonstrated that exercise can modulate Kupffer cell polarization towards an anti-inflammatory phenotype, prompting further investigation into whether ZLN005 similarly influences immune cell phenotype within the circulation and bone marrow. Furthermore, the use of DMSO as a ZLN005 vehicle for intraperitoneal injection necessitates careful consideration due to its inherent cytotoxic properties. Future studies should explore alternative delivery methods to mitigate potential confounding effects of DMSO. Besides these limitations, the present study demonstrates that a 3-day pretreatment with ZLN005 effectively upregulates hepatocyte mitochondrial biogenesis and attenuates liver IRI and hepatic tumor metastases.

## 5. Conclusions

This study reveals the novel finding that a brief course of intraperitoneal ZLN005 administration in mice provides significant protection against liver IRI- and I/R-induced metastases. Furthermore, both in vivo and in vitro experiments demonstrate that ZLN005 directly upregulates mitochondrial biogenesis in hepatocytes. These findings have important clinical implications, suggesting that preoperative treatment with ZLN005 could be a powerful and innovative strategy to mitigate liver IRI, improve short-term postoperative hepatic function, and enhance long-term oncologic outcomes.

## Data Availability

No new data sets were created.

## Supplementary Materials

The following supporting information can be downloaded at: https://www.mdpi.com/article/10.3390/cells13171448/s1, Figure S1: Assessment of Liver Injury after ZLN005 pretreatment. Figure S2: Quantitative analysis of COXIV staining in MHC and HHC.

## References

[1] Martin J., Petrillo A., Smyth E.C., Shaida N., Khwaja S., Cheow H., Duckworth A., Heister P., Praseedom R., Jah A. (2020). Colorectal Liver Metastases: Current Management and Future Perspectives. World J. Clin. Oncol..

[2] Wu C.-C., Yeh D.-C., Ho W.-M., Yu C.-L., Cheng S.-B., Liu T.-J., P’eng F.-K. (2002). Occlusion of Hepatic Blood Inflow for Complex Central Liver Resections in Cirrhotic Patients: A Randomized Comparison of Hemihepatic and Total Hepatic Occlusion Techniques. Arch. Surg..

[3] Choi E.K., Lim D.G. (2022). Hepatic Ischemia-Reperfusion Injury with Respect to Oxidative Stress and Inflammatory Response: A Narrative Review. J. Yeungnam Med. Sci..

[4] Creasy J.M., Sadot E., Koerkamp B.G., Chou J.F., Gonen M., Kemeny N.E., Balachandran V.P., Kingham T.P., DeMatteo R.P., Allen P.J. (2018). Actual 10-Year Survival after Hepatic Resection of Colorectal Liver Metastases: What Factors Preclude Cure?. Surgery.

[5] Peralta C., Jiménez-Castro M.B., Gracia-Sancho J. (2013). Hepatic Ischemia and Reperfusion Injury: Effects on the Liver Sinusoidal Milieu. J. Hepatol..

[6] Kaltenmeier C., Wang R., Popp B., Geller D., Tohme S., Yazdani H.O. (2022). Role of Immuno-Inflammatory Signals in Liver Ischemia-Reperfusion Injury. Cells.

[7] Huang H., Tohme S., Al-Khafaji A.B., Tai S., Loughran P., Chen L., Wang S., Kim J., Billiar T., Wang Y. (2015). DAMPs-Activated Neutrophil Extracellular Trap Exacerbates Sterile Inflammatory Liver Injury. Hepatology.

[8] Kaltenmeier C., Yazdani H.O., Handu S., Popp B., Geller D., Tohme S. (2022). The Role of Neutrophils as a Driver in Hepatic Ischemia-Reperfusion Injury and Cancer Growth. Front. Immunol..

[9] Tohme S., Kameneva M.V., Yazdani H.O., Sud V., Goswami J., Loughran P., Huang H., Simmons R.L., Tsung A. (2017). Drag Reducing Polymers Decrease Hepatic Injury and Metastases after Liver Ischemia-Reperfusion. Oncotarget.

[10] Yazdani H.O., Kaltenmeier C., Morder K., Moon J., Traczek M., Loughran P., Zamora R., Vodovotz Y., Li F., Wang J.H.-C. (2021). Exercise Training Decreases Hepatic Injury via Changes in Immune Response to Liver Ischemia/Reperfusion in Mice. Hepatology.

[11] van der Windt D.J., Sud V., Zhang H., Tsung A., Huang H. (2018). The Effects of Physical Exercise on Fatty Liver Disease. Gene Expr..

[12] Sheinboim D., Parikh S., Manich P., Markus I., Dahan S., Parikh R., Stubbs E., Cohen G., Zemser-Werner V., Bell R.E. (2022). An Exercise-Induced Metabolic Shield in Distant Organs Blocks Cancer Progression and Metastatic Dissemination. Cancer Res..

[13] Warren J.L., Hunter G.R., Gower B.A., Bamman M.M., Windham S.T., Moellering D.R., Fisher G. (2020). Exercise Effects on Mitochondrial Function and Lipid Metabolism during Energy Balance. Med. Sci. Sports Exerc..

[14] Lima F.D., Stamm D.N., Della-Pace I.D., Dobrachinski F., de Carvalho N.R., Royes L.F.F., Soares F.A., Rocha J.B., González-Gallego J., Bresciani G. (2013). Swimming Training Induces Liver Mitochondrial Adaptations to Oxidative Stress in Rats Submitted to Repeated Exhaustive Swimming Bouts. PLoS ONE.

[15] Stevanović J., Beleza J., Coxito P., Ascensão A., Magalhães J. (2019). Physical Exercise and Liver “Fitness”: Role of Mitochondrial Function and Epigenetics-Related Mechanisms in Non-Alcoholic Fatty Liver Disease. Mol. Metab..

[16] Zhang L.-N., Zhou H.-Y., Fu Y.-Y., Li Y.-Y., Wu F., Gu M., Wu L.-Y., Xia C.-M., Dong T.-C., Li J.-Y. (2013). Novel Small-Molecule PGC-1α Transcriptional Regulator With Beneficial Effects on Diabetic Db/Db Mice. Diabetes.

[17] Xu Y., Kabba J.A., Ruan W., Wang Y., Zhao S., Song X., Zhang L., Li J., Pang T. (2018). The PGC-1α Activator ZLN005 Ameliorates Ischemia-Induced Neuronal Injury In Vitro and In Vivo. Cell. Mol. Neurobiol..

[18] Wang Z., Fu Z., Wang C., Xu J., Ma H., Jiang M., Xu T., Feng X., Zhang W. (2021). ZLN005 Protects against Ischemia-Reperfusion-Induced Kidney Injury by Mitigating Oxidative Stress through the Restoration of Mitochondrial Fatty Acid Oxidation. Am. J. Transl. Res..

[19] Yazdani H.O., Tohme S. (2019). Murine Model of Metastatic Liver Tumors in the Setting of Ischemia Reperfusion Injury. J. Vis. Exp. JoVE.

[20] Tsung A., Hoffman R.A., Izuishi K., Critchlow N.D., Nakao A., Chan M.H., Lotze M.T., Geller D.A., Billiar T.R. (2005). Hepatic Ischemia/Reperfusion Injury Involves Functional TLR4 Signaling in Nonparenchymal Cells1. J. Immunol..

[21] Tohme S., Yazdani H.O., Sud V., Loughran P., Huang H., Zamora R., Simmons R.L., Vodovotz Y., Tsung A. (2019). Computational Analysis Supports IL-17A as a Central Driver of Neutrophil Extracellular Trap-Mediated Injury in Liver Ischemia Reperfusion. J. Immunol..

[22] Charni-Natan M., Goldstein I. (2020). Protocol for Primary Mouse Hepatocyte Isolation. STAR Protoc..

[23] Rooney J., Ryde I., Sanders L., Howlett E., Colton M., Germ K., Mayer G., Greenamyre J., Meyer J. (2015). PCR Based Determination of Mitochondrial DNA Copy Number in Multiple Species. Methods Mol. Biol..

[24] Kaltenmeier C., Yazdani H., Molinari M., Simmons R., Geller D., Tohme S. (2021). The Role of Exercise Training in Preconditioning the Liver against Ischemia/Reperfusion Injury in an Orthotopic Liver Murine Transplant Model. HPB.

[25] Arhin N.D., Shen C., Bailey C.E., Matsuoka L.K., Hawkins A.T., Holowatyj A.N., Ciombor K.K., Hopkins M.B., Geiger T.M., Kam A.E. (2021). Surgical Resection and Survival Outcomes in Metastatic Young Adult Colorectal Cancer Patients. Cancer Med..

[26] Machado I.F., Palmeira C.M., Rolo A.P. (2023). Preservation of Mitochondrial Health in Liver Ischemia/Reperfusion Injury. Biomedicines.

[27] Rossmann M.P., Dubois S.M., Agarwal S., Zon L.I. (2021). Mitochondrial Function in Development and Disease. Dis. Model. Mech..

[28] Adzigbli L., Sokolov E.P., Wimmers K., Sokolova I.M., Ponsuksili S. (2022). Effects of Hypoxia and Reoxygenation on Mitochondrial Functions and Transcriptional Profiles of Isolated Brain and Muscle Porcine Cells. Sci. Rep..

[29] Azimifar S.B., Nagaraj N., Cox J., Mann M. (2014). Cell-Type-Resolved Quantitative Proteomics of Murine Liver. Cell Metab..

[30] Martins R.M., Teodoro J.S., Furtado E., Rolo A.P., Palmeira C.M., Tralhão J.G. (2019). Evaluation of Bioenergetic and Mitochondrial Function in Liver Transplantation. Clin. Mol. Hepatol..

[31] Kolios G., Valatas V., Kouroumalis E. (2006). Role of Kupffer Cells in the Pathogenesis of Liver Disease. World J. Gastroenterol. WJG.

[32] Deshmane S.L., Kremlev S., Amini S., Sawaya B.E. (2009). Monocyte Chemoattractant Protein-1 (MCP-1): An Overview. J. Interferon Cytokine Res..

[33] Nairz M., Sonnweber T., Schroll A., Theurl I., Weiss G. (2012). The Pleiotropic Effects of Erythropoietin in Infection and Inflammation. Microbes Infect. Inst. Pasteur.

[34] Jiao Q., Xiang L., Chen Y. (2024). Mitochondrial Transplantation: A Promising Therapy for Mitochondrial Disorders. Int. J. Pharm..

[35] Cruz-Gregorio A., Aranda-Rivera A.K., Amador-Martinez I., Maycotte P. (2023). Mitochondrial Transplantation Strategies in Multifaceted Induction of Cancer Cell Death. Life Sci..

[36] Ulger O., Kubat G.B. (2022). Therapeutic Applications of Mitochondrial Transplantation. Biochimie.

[37] Wang C., Li Z., Zhao B., Wu Y., Fu Y., Kang K., Li Y., Dong L., Li X., Zhang B. (2021). PGC-1α Protects against Hepatic Ischemia Reperfusion Injury by Activating PPARα and PPARγ and Regulating ROS Production. Oxid. Med. Cell. Longev..

[38] Yuan Y., Tian Y., Jiang H., Cai L., Song J., Peng R., Zhang X. (2023). Mechanism of PGC-1α-Mediated Mitochondrial Biogenesis in Cerebral Ischemia–Reperfusion Injury. Front. Mol. Neurosci..

[39] Li Y., Jiao Y., Liu Y., Fu J., Sun L., Su J. (2022). PGC-1α Protects from Myocardial Ischaemia-reperfusion Injury by Regulating Mitonuclear Communication. J. Cell. Mol. Med..

[40] Wang D., Cao L., Zhou X., Wang G., Ma Y., Hao X., Fan H. (2022). Mitigation of Honokiol on Fluoride-Induced Mitochondrial Oxidative Stress, Mitochondrial Dysfunction, and Cognitive Deficits through Activating AMPK/PGC-1α/Sirt3. J. Hazard. Mater..

[41] Abu Shelbayeh O., Arroum T., Morris S., Busch K.B. (2023). PGC-1α Is a Master Regulator of Mitochondrial Lifecycle and ROS Stress Response. Antioxidants.

[42] Li C.X., Ling C.C., Shao Y., Xu A., Li X.C., Ng K.T.-P., Liu X.B., Ma Y.Y., Qi X., Liu H. (2016). CXCL10/CXCR3 Signaling Mobilized-Regulatory T Cells Promote Liver Tumor Recurrence after Transplantation. J. Hepatol..

[43] Maspero M., Yilmaz S., Cazzaniga B., Raj R., Ali K., Mazzaferro V., Schlegel A. (2023). The Role of Ischaemia-Reperfusion Injury and Liver Regeneration in Hepatic Tumour Recurrence. JHEP Rep..

[44] Fnu G., Weber G.F. (2023). Osteopontin Induces Mitochondrial Biogenesis in Deadherent Cancer Cells. Oncotarget.

[45] Bai R., Cui J. (2023). Mitochondrial Immune Regulation and Anti-Tumor Immunotherapy Strategies Targeting Mitochondria. Cancer Lett..

[46] Li W., Li X., Wang B., Chen Y., Xiao A., Zeng D., Ou D., Yan S., Li W., Zheng Q. (2016). ZLN005 Protects Cardiomyocytes against High Glucose-Induced Cytotoxicity by Promoting SIRT1 Expression and Autophagy. Exp. Cell Res..

[47] Zhu P., Ma H., Cui S., Zhou X., Xu W., Yu J., Li J. (2022). ZLN005 Alleviates In Vivo and In Vitro Renal Fibrosis via PGC-1α-Mediated Mitochondrial Homeostasis. Pharmaceuticals.

[48] Suzuki Y., Kami D., Taya T., Sano A., Ogata T., Matoba S., Gojo S. (2023). ZLN005 Improves the Survival of Polymicrobial Sepsis by Increasing the Bacterial Killing via Inducing Lysosomal Acidification and Biogenesis in Phagocytes. Front. Immunol..

[49] Maurice N.M., Bedi B., Yuan Z., Lin K.-C., Goldberg J.B., Hart C.M., Bailey K.L., Sadikot R.T. (2022). The Effect of PGC-1alpha-SIRT3 Pathway Activation on Pseudomonas Aeruginosa Infection. Pathogens.

