# Supplementary material for: ZLN005, a PGC-1α Activator, Protects the Liver against Ischemia–Reperfusion Injury and the Progression of Hepatic Metastases
Source: Cells. 2024 Aug 29;13(17):1448. doi: 10.3390/cells13171448 (PMC11393917; doi:10.3390/cells13171448)
Supplement: Supplementary file 1 [file cells-13-01448-s001.zip › cells-3159006-supplementary.pdf]

ZLN005, a PGC-1α Activator, Protects the Liver against Ischemia–Reperfusion Injury and the Progression of Hepatic Metastases

Celine Tohme [1,†], Tony Haykal [1,†], Ruiqi Yang [1,2], Taylor J. Austin [1], Patricia Loughran [1,3], David A. Geller [1], Richard L. Simmons [1], Samer Tohme [1,*] and Hamza O. Yazdani [1,*]

1 Department of Surgery, University of Pittsburgh School of Medicine, Pittsburgh, PA 15213, USA; tohmec@upmc.edu (C.T.); haykalt@upmc.edu (T.H.); ruiqiy@upmc.edu (R.Y.); austint4@upmc.edu (T.J.A.); loughranp@upmc.edu (P.L.); gellerda@upmc.edu (D.A.G.); simmonsrl@upmc.edu (R.L.S.)
2 School of Medicine, Tsinghua University, Beijing 100084, China
3 Center for Biologic Imaging, Department of Cell Biology, University of Pittsburgh, Pittsburgh, PA 15213, USA
* Correspondence: tohmest@upmc.edu (S.T.); obaidh3@upmc.edu (H.O.Y.)
† These authors contributed equally to this work.

Supplementary Figure

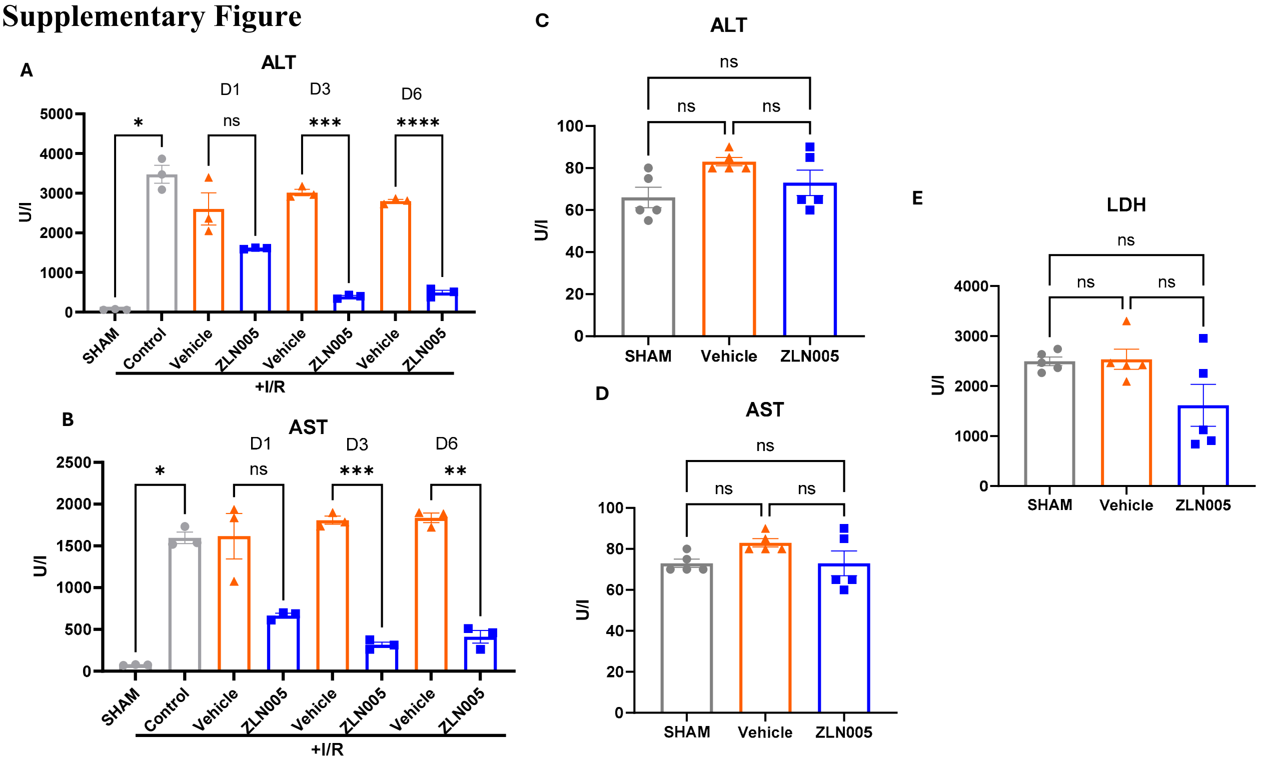

Figure S1. Assessment of Liver Injury after ZLN005 pre-treatment. **(A)** Serum ALT was measured after 1 hour of ischemia and 6 hours of reperfusion. **(B)** Serum AST was measured after 1 hour of ischemia and 6 hours of reperfusion. **(C)** Serum ALT was measured after 3 days of ZLN005 pretreatment. **(D)** Serum AST was measured after 3 days of ZLN005 pretreatment. **(E)** Serum LDH was measured after 3 days of ZLN005 pretreatment.

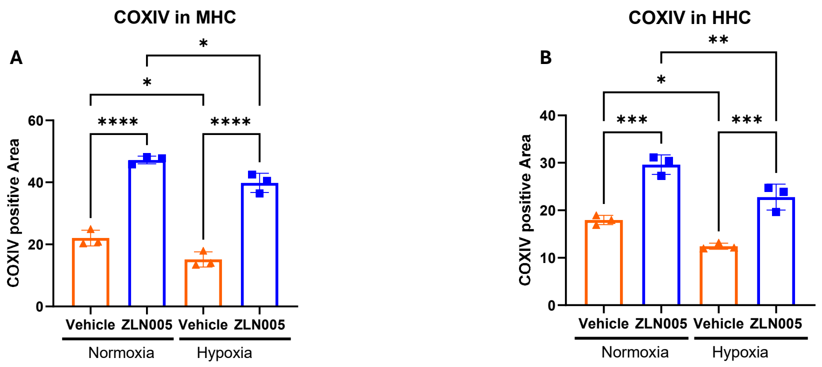

Figure S2. Quantitative analysis of COXIV staining in MHC and HHC. **(A)** Quantitative analysis of COXIV staining in MHC under normoxic and hypoxic conditions. **(B)** Quantitative analysis of COXIV staining in HHC under normoxic and hypoxic conditions.
